# Diagnostic testing and antibiotic stewardship for pneumonia in children worldwide: current developments and next steps

**DOI:** 10.1097/MOP.0000000000001484

**Published:** 2025-09-04

**Authors:** Josephine S. van de Maat, Andrew Redfern, Tigist Bacha, Jeroen Schouten, Esmée Ruizendaal

**Affiliations:** a Department of Pediatrics, Amalia Children's Hospital; b Radboud Community for Infectious Diseases; c Department of Laboratory Medicine, Laboratory of Medical Immunology, Radboud University Medical Center, Nijmegen, Netherlands; d Department of Pediatrics & Child Health, Faculty Medicine & Health Sciences, Stellenbosch University, Cape Town, South Africa; e Department of Pediatrics and Child Health, Saint Paul, Millennium Medical College, Addis Ababa, Ethiopia; f Department of Intensive Care; g Department of Medical Microbiology, Radboud University Medical Center, Nijmegen, the Netherlands

**Keywords:** antimicrobial stewardship, bacterial infection, diagnostic tests, pneumonia, respiratory tract infections

## Abstract

**Purpose of review:**

Routine diagnostic tests for childhood pneumonia lack the accuracy to identify bacterial pneumonia, leading to inappropriate antibiotic prescription. Novel tests are being developed. Optimizing diagnostic strategies using available diagnostic tools and exploring the role of new tests is essential to improve antimicrobial stewardship (AMS) in children. This review provides an overview of advances in diagnostic testing for pediatric pneumonia and discusses how strategies can be optimized in different settings in order to improve AMS.

**Recent findings:**

All currently available tests for bacterial pneumonia are limited in their diagnostic accuracy. However, in settings with high baseline antibiotic prescription, routine diagnostics such as CRP or PCT-guided prescription can improve antibiotic use. Among the innovative tests, lung ultrasound with computer-aided detection and prediction models combining multiple tests holds most promise for low-resource settings. For high-resource settings, RNA signatures and next-generation sequencing are promising developments. The impact of innovative tests on AMS remains to be evaluated.

**Summary:**

Robust diagnostic and contextual research is needed to develop new diagnostic tests and to optimize current strategies for bacterial pneumonia in children. In order to tailor diagnostic approaches to specific settings, broad impact studies and stratification of risk groups are crucial.

## INTRODUCTION

Lower respiratory tract infections (RTIs) are the leading cause of hospital admissions and death in children under five globally [1]. Despite most RTIs being viral, they are the primary reason for antibiotic prescriptions in children. Targeting antibiotics appropriately is crucial for improving health outcomes and combating antimicrobial resistance (AMR). However, current diagnostic tests lack the accuracy to identify bacterial pneumonia in children, and test availability varies across settings [2]. Additionally, approaches like the Integrated Management of Childhood Illness do not distinguish between bacterial and viral pneumonia, prioritizing sensitivity over specificity [3]. Optimizing diagnostic strategies for lower RTIs, using available diagnostic tools and exploring new tests, is essential to improve antimicrobial stewardship (AMS) in children. This review provides an overview of advances in diagnostic testing for pediatric pneumonia and discusses how strategies can be optimized in both low- and high-resource settings in order to improve AMS, with a focus on outpatient care. 

**Box 1:**
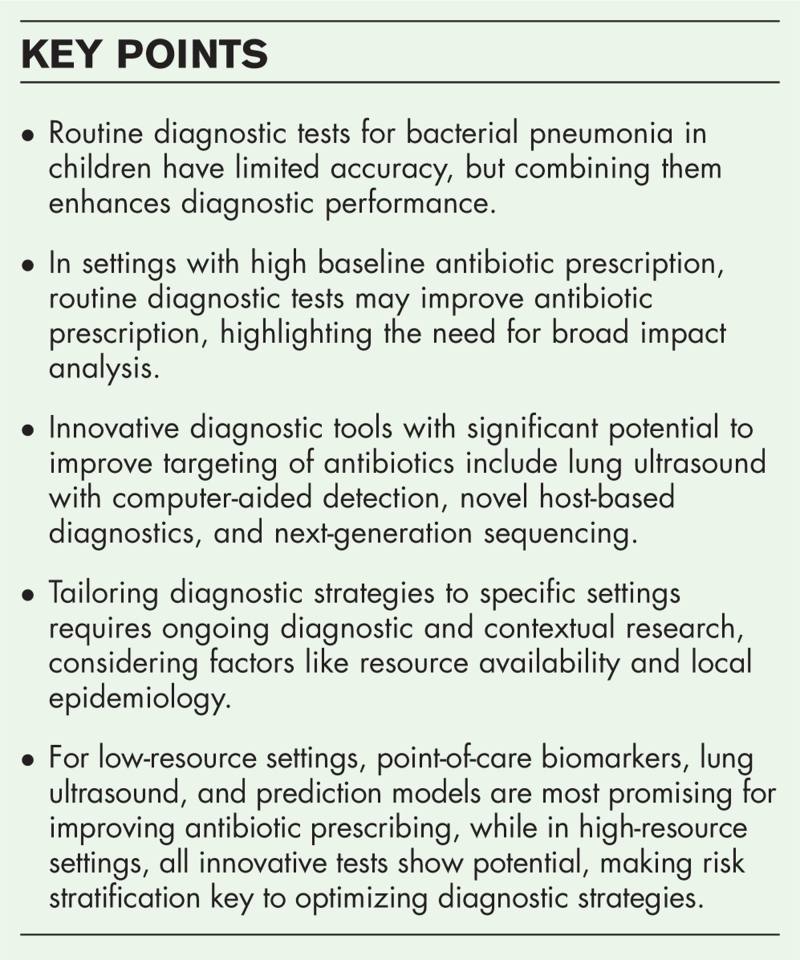
no caption available

## WHAT IS THE IDEAL DIAGNOSTIC TEST FOR CHILDHOOD PNEUMONIA?

**FIGURE 1:**
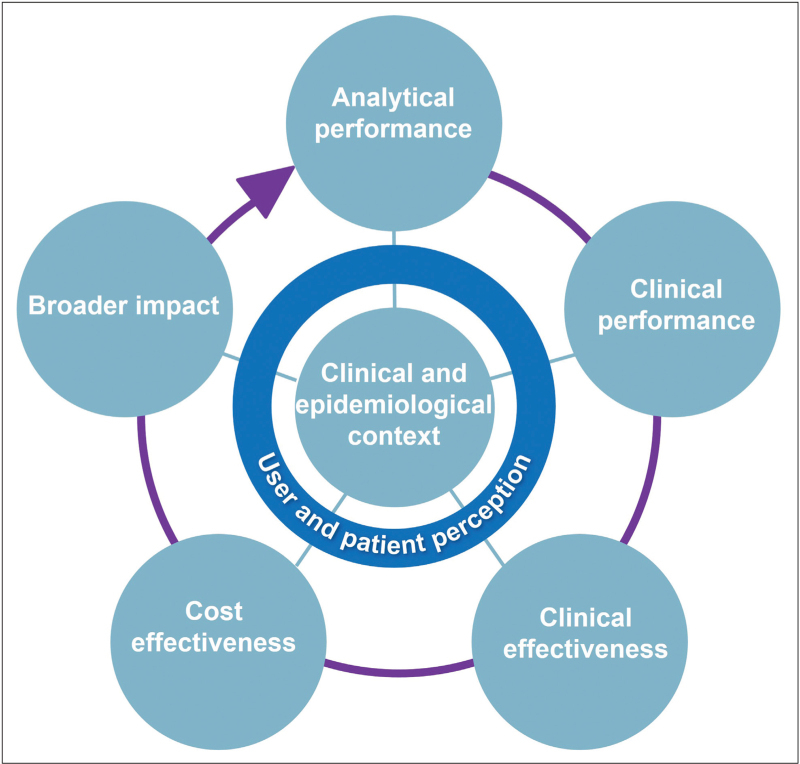
Framework for the development and evaluation of diagnostic tests (adapted from [4,5]).

Many diagnostic tests are available for childhood pneumonia, raising questions about optimal strategies. Figure 1 shows a framework (adapted from [4,5]) to evaluate tests across different evidence levels, always considering the local clinical and epidemiological context. The first component is analytical performance, or how well a test meets predefined quality standards (e.g. diagnostic accuracy) in the laboratory or in the development phase. WHO target product profiles (TPPs) suggest that diagnostic tests discriminating between bacterial and nonbacterial infections should have more than 90% sensitivity, more than 80% specificity, rapid turnaround time (<10 min ideally), flexible storage, and minimal sampling needs [6]. The second component is clinical performance, meaning diagnostic accuracy in clinical practice. Tests must be validated in different resource and healthcare levels across diverse settings, as factors like malnutrition, HIV, and vaccination status may affect their performance [7]. Third is clinical effectiveness, or real-world impact on relevant health outcomes, such as safety and antibiotic prescription. A key goal is to safely reduce antibiotic overuse through ‘timely and accurate diagnosis’, a priority identified by the WHO to combat AMR [8,9^▪^]. Contextual differences such as local baseline prescription and infection rates will influence effectiveness. Cost-effectiveness is the fourth component, assessing patient benefit relative to healthcare and societal costs. Lastly, the fifth component considers the broader impact a test may have on public health, AMR reduction and healthcare systems, although this is often difficult to evaluate.

## CURRENT AND UPCOMING DIAGNOSTIC TESTS AND THEIR VALUE IN ANTIMICROBIAL STEWARDSHIP

In light of the presented framework, we will discuss the current and upcoming diagnostic tests for pediatric pneumonia, each with its own advantages and limitations. Table 1 provides an overview of both routine tests and more innovative diagnostic methods, along with their performance and impact on antibiotic prescription.

**Table 1:**
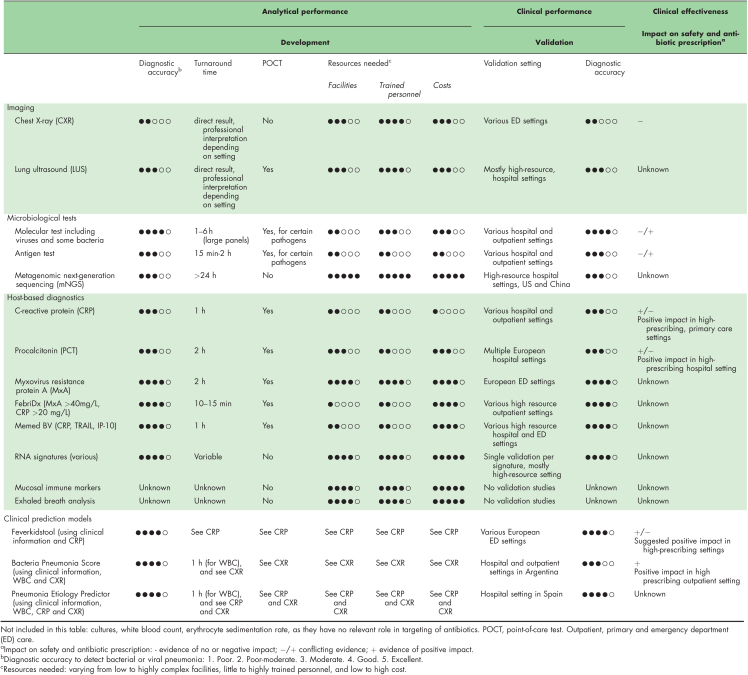
Overview of diagnostic tests for childhood pneumonia and their role in targeting of antibiotics

## IMAGING

Chest X-rays (CXR) have traditionally been used as the reference standard for diagnosing pneumonia. However, they have several important limitations, most importantly their poor to moderate interobserver agreement and an inability to differentiate between viral and bacterial infections [10–12]. In addition, CXR interpretation requires radiological training that is often absent in low-resource and primary care settings. Studies have shown that CXR results rarely impact the decision to prescribe antibiotics [13,14].

Point-of-care lung ultrasound (LUS) has been shown to have higher sensitivity and better negative predictive value for pneumonia compared to CXR, although the majority of studies come from high resource settings [15^▪▪^,^16^]. LUS features may distinguish viral from bacterial causes and therefore has the potential to reduce unnecessary antibiotic use [17–20], although there remains heterogeneity in LUS reporting practice and definitions of bacterial pneumonia [21]. Still, combining a biomarker (procalcitonin, PCT) with suggestive LUS or CXR features improved diagnostic accuracy for bacterial pneumonia, with LUS/PCT being superior to CXR/PCT [22^▪^]. A diagnostic algorithm combining LUS/PCT did not result in any cases of bacterial pneumonia being mistreated, and would have resulted in reducing unnecessary antibiotics compared to CXR/PCT [22^▪^]. Despite showing such promise, studies demonstrating the effectiveness of LUS to reduce antibiotic use are lacking.

Computer-aided detection (CAD) shows promise for standardizing imaging interpretation and is widely used for diagnosing adult tuberculosis [23]. However, it is not yet suitable for pediatric pneumonia. Two systematic reviews of CAD with CXR as the reference found high performance, but most included studies relied on a single public dataset, were retrospective, lacked external validation, and did not compare results with clinician assessments, limiting generalizability and clinical applicability [24,25]. Further development and validation are needed for pediatric use.

## MICROBIOLOGICAL TESTING

Several microbiological tests are available that can aid in detecting the causative pathogen of pneumonia, with bacterial sputum culture being used most frequently. However, in childhood pneumonia, their role is very limited. Sputum is difficult to obtain from children and even cultures of induced sputum have limited diagnostic accuracy. Therefore, they are rarely useful in clinical practice [26,27]. Blood cultures are typically negative in RTIs and rarely lead to changes in antibiotic treatment [28–30]. Additionally, in primary care or resource-limited settings, microbiology laboratories may not be available, or the prolonged turn-around-time limits clinical utility.

Rapid viral testing, using antigen or molecular methods, is now widely available in both high- and low-resource settings [31]. These tests range from single-virus tests [e.g. influenza or respiratory syncytial virus (RSV)] to large syndromic PCR panels detecting multiple viruses, atypical bacteria, and bacterial resistance genes [32,33]. While frequently used, the impact of viral tests on antibiotic prescribing for respiratory infections is inconsistent. Some nonrandomized studies report reduced antibiotic prescription, but randomized trials have not demonstrated this effect [34–37]. A key limitation is that a positive viral result does not exclude bacterial co-infection. Syndromic PCR panels including common bacterial pathogens may help, but challenges remain, such as differentiating colonization from infection and limited concordance between upper and lower respiratory tract. A recent trial in ICU patients studied the impact of a large panel on AMS and clinical outcomes [38]. The test showed modest AMS benefits, but failed to prove noninferiority for clinical outcomes, despite strong diagnostic accuracy in the same setting. Although the ICU differs from our focus area, these results highlight that even highly accurate diagnostics may not translate into adequate clinical effectiveness.

Recent developments in microbiological testing include metagenomic next-generation sequencing (mNGS). mNGS enables simultaneous detection of nearly all pathogens in a sample without predefining targets and can identify AMR. It outperforms conventional culture in detecting respiratory pathogens in bronchoalveolar lavage samples [39,40]. However, its high sensitivity can complicate interpretation due to colonizing bacteria or contamination from reagents and the environment. Targeted NGS (tNGS) may address this by selectively enriching relevant pathogen sequences, though its role in clinical practice remains uncertain [41,42]. The broader use of NGS is currently limited by long turnaround times, high costs, and the need for specialized equipment and staff. As a potential solution, artificial intelligence could help automate result analysis and interpretation, potentially improving accessibility and utility in clinical settings [43].

## HOST-BASED DIAGNOSTICS

Blood-based host-protein biomarkers are more widely available and commonly used in clinical practice, though their performance varies. C-reactive protein (CRP) and procalcitonin (PCT) have moderate diagnostic accuracy, while white blood count and erythrocyte sedimentation rate perform poorly [44–46]. CRP is the most widely available and well evaluated test, also for use as a point-of-care test (POCT). Cut-offs vary greatly across studies, mostly from more than 20 mg/l to more than 80 mg/l. PCT use is more limited and highly variable, with cut-offs used ranging from more than 0.5 ng/ml to more than 2 ng/mL [46]. While none of these biomarkers are accurate enough to distinguish between viral and bacterial causes on their own, some studies have shown a well tolerated reduction in antibiotic prescriptions in primary care when CRP-guided prescribing is implemented [47^▪▪^,^48^–^50^], but others found no impact [51–53]. Findings are similar for PCT-guided prescription [54,55]. The interventions had most impact when baseline prescription rates were high.

New host-based viral markers like myxovirus resistance protein A (MxA) and tumor necrosis factor-related apoptosis-inducing ligand (TRAIL) have recently been developed, often in combination with bacterial markers [56–58]. FebriDx is POCT that detects elevated concentrations of MxA (>40 mg/l) and CRP (>20 mg/l) in a qualitative manner, showing promising diagnostic accuracy. One study evaluated the intention to prescribe antibiotics before and after the FebriDx result, resulting in a lower intention to prescribe, but its actual impact on prescription rates still needs to be demonstrated [59^▪^,^60^,^61^]. Given the low cut-off for CRP, improved targeting of antibiotics can be expected especially in settings with high baseline prescription. The role of MxA alone is probably limited, as a high concentration does not exclude bacterial co-infection.

Another novel host-protein-based test, Memed BV or ImmunoXpert, combines CRP, TRAIL, and interleukin 6 (IL-6) to produce a score that classifies the infection's etiology as viral (score 0–34), equivocal (35–65), or bacterial (66–100) [62]. This test has been widely validated and demonstrates high diagnostic accuracy for distinguishing bacterial versus viral infections [63–66,67^▪▪^]. It outperforms routine tests like CRP and PCT, with a smaller “grey area” of uncertain etiology compared to CRP alone. Given this diagnostic accuracy, its potential impact on antibiotic prescription is promising, but has not yet been studied. Several other combinations of host-based proteins have been proposed, so far without external validation or impact studies [44,68].

A promising recent development in blood biomarkers is the use of RNA signatures, combinations of genes that are differentially expressed in health and disease. Specific gene expression patterns have been identified for infectious diseases, allowing for the differentiation between viral and bacterial infections, tuberculosis, and malaria [69–71]. Two RNA signatures have been developed to distinguish between bacterial and viral pneumonia in children, with one specifically designed for malaria-endemic countries [72^▪▪^,^73^]. These signatures demonstrate very high diagnostic accuracy, making them a promising tool for the future diagnosis and treatment of childhood pneumonia. While they are currently only available in research settings, efforts are underway to prepare for their integration into routine care, including combining different assays into a single platform and developing point-of-care technology [74,75^▪▪^].

Lastly, two innovative developments are mucosal immune markers and exhaled breath analysis. These methods are noninvasive, easy to collect, and sample directly from the respiratory tract. Immune markers in nasal mucosa and saliva have been linked to RSV in adults [76] and severe pneumonia in children [77–79]. However, more research is needed to assess their ability to differentiate bacterial from viral RTIs. Exhaled breath analysis detects volatile organic compounds from inflammatory and metabolic processes. Current evidence for exhaled breath analysis is in specific risk groups [80], but it may have future potential for broader RTI groups [81,82].

## CLINICAL PREDICTION MODELS

Clinical prediction models (CPMs) offer another diagnostic approach: algorithms predicting the likelihood of disease, usually based on a combination of clinical data with diagnostic test results. Various CPMs have been developed for childhood pneumonia, although they are rarely used in routine care. The Feverkidstool combines 10 clinical parameters and CRP to predict bacterial pneumonia or other serious bacterial infections [83]. It has shown good performance across various settings, including low-prevalence and immunocompromised populations [84–88,89^▪^]. A Dutch study found no overall reduction in antibiotic use but improved targeting of antibiotics [90], while a European simulation study suggested its potential to reduce prescriptions in high-use and low-risk groups [91].

The Bacterial Pneumonia Score (BPS) uses blood biomarkers and CXR to differentiate bacterial from viral infections [92]. Developed in Argentina, it showed high diagnostic accuracy in hospitalized children, though performance declined in validation studies and a study with a simplified BPS version [93,94]. Still, in outpatient settings, it was well tolerated [95] and significantly reduced antibiotic use (47 versus 87%) [96].

The Pneumonia Etiology Predictor uses a two-step approach – using clinical, laboratory, and CXR data – to differentiate viral from bacterial pneumonia, demonstrating strong performance in Spanish hospitals [97].

Overall, CPMs vary in complexity but show potential, with the Feverkidstool and BPS demonstrating improved antibiotic stewardship [90,96].

## SUMMARY AND NEXT STEPS

Looking at the current landscape and the horizon of diagnostic tests for childhood pneumonia: what are the gaps, how to fill these and what are the most promising diagnostics for different settings?

## CURRENT EVIDENCE GAPS AND IMPLICATIONS FOR RESEARCH

All currently available tests for bacterial pneumonia have limited diagnostic accuracy and no strategy meets the TPP. To improve quality of diagnostic and antibiotic use, new tests must be developed, but optimizing diagnostic strategies with the current repertoire and implementation in clinical practice are equally crucial. This requires robust *diagnostic research*, covering all components of Fig. 1. Many tests are developed and validated in single settings, but broad validation and – most importantly – impact studies in different settings and risk groups are crucial. Research should focus on outpatient settings (primary care and emergency department), where most decisions on initiation of antibiotics are made. These settings highlight the importance of short turnaround times and point-of-care tests. Combining diagnostic methods, such as LUS with PCT or PCT with clinical parameters, may enhance diagnostic accuracy and antibiotic prescription [22^▪^,89^▪^].

As shown before, also nonideal tests can safely reduce antibiotic prescription in certain settings, for example, CRP-guided prescription in primary care in Vietnam [48], and a moderately performing prediction model in the outpatient setting in Argentina [96]. This highlights the need for *contextual research*, as shown in the center of Fig. 1. The clinical and epidemiological context includes factors like baseline infection prevalence, comorbidities, baseline antibiotic prescription rates, healthcare factors, and level of resources. In addition, knowing the patient and user perception is essential for effective implementation of a diagnostic intervention. This area of research, known as implementation science, goes beyond the scope of this review.

## PROMISING STRATEGIES FOR IMPROVING ANTIBIOTIC PRESCRIPTION IN DIFFERENT SETTINGS

A major difference between settings is resource availability. In low-resource settings, diagnostic strategies should be simple, affordable and require minimal infrastructure, while integrating advanced technologies like artificial intelligence. Therefore, point-of-care tests are most promising. Many low-resource settings not only have high antibiotic use but also risk factors for severe infections such as HIV and malnutrition. Low-cost interventions like CRP-guided prescription or the Feverkidstool should be evaluated in these settings. Among innovative tests, LUS, with or without biomarkers and combined with CAD, shows promise, and is currently being evaluated in a trial in Africa [98]. An advantage of LUS is that it also potentially provides diagnostic information on other relevant diagnoses like pulmonary tuberculosis [99]. In the future, multiclass RNA signatures could be valuable if made scalable and affordable.

High-resource settings with low rates of serious infections should focus on risk stratification, saving novel tests for patient groups with most diagnostic uncertainty. Primary care, where most antibiotics are prescribed, remains understudied [100]. Here, point-of-care tests could reduce unnecessary antibiotic use, especially when prescribing rates are high. Simultaneously, de-implementing low-impact or unnecessary diagnostics is essential. High-resource settings also provide a strong foundation for developing and testing innovative diagnostics that could later be adapted for use in low-resource environments.

## CONCLUSION

Accurate diagnostic tools for bacterial pneumonia in children that can guide antibiotic prescription are urgently needed. Diagnostic research is crucial to develop new tests and to optimize strategies across various settings. Depending on resources, epidemiology and local factors, diagnostic strategies for childhood pneumonia can be adapted. In low-resource settings, point-of-care biomarkers, LUS, or prediction models combining these tests are most promising. In high-resource settings, all innovative tests show potential, so risk stratification is crucial to tailor diagnostic strategies appropriately.

## Acknowledgements

*None.*

### Financial support and sponsorship

*J.vd.M. was supported by a two early career grants: a Springboard award of the European Society of Paediatric Infectious Diseases (ESPID) and a TULIPS – Auxilium & Caritas award.*

### Conflicts of interest

*There are no conflicts of interest.*

## REFERENCES AND RECOMMENDED READING

Papers of particular interest, published within the annual period of review, have been highlighted as:

▪ of special interest
▪▪ of outstanding interest
